# Courage is not the absence of fear: responding to the Ebola outbreak in Liberia

**DOI:** 10.9745/GHSP-D-14-00157

**Published:** 2014-12-02

**Authors:** Linda Meta Mobula

**Affiliations:** a United States Agency for International Development/AAAS S&T Fellow, Washington, DC, USA, and the Johns Hopkins Bloomberg School of Public Health, Baltimore, MD, USA. While in Liberia, with the Disaster Assistance Response Team (DART) of Samaritan's Purse.

Since the onset of the Ebola outbreak in West Africa in February 2014, the World Health Organization estimates that more than 370 health care workers have been infected with Ebola virus disease (EVD), and 216 have since died.^1^

During the height of the Ebola epidemic, I had the unique opportunity of working at the Ebola Case Management Center of the Eternal Love Winning Africa (ELWA) hospital in Monrovia, Liberia. About a third of all patients with Ebola admitted to ELWA were health care workers.^2^ Some of these brave individuals had worked tirelessly, treating Ebola patients while fully aware that they could become infected themselves. Others were likely exposed to EVD at their respective government health facilities, unaware that their patients had Ebola. Exposure to Ebola patients outside of an Ebola management center increases one's risk of contracting the illness, especially since there are no infection control measures in place for protection.

A famous quote from author Ambrose Hollingworth Redmoon (born James Neil Hollingworth) goes^3^:

Courage is not the absence of fear, but rather the judgment that something else is more important than fear. The timid presume it is lack of fear that allows the brave to act when the timid do not. But to take action when one is not afraid is easy. To refrain when afraid is also easy. To take action regardless of fear is brave.

Redmoon's assertion describes perfectly the bravery of those who have sacrificed their lives to treat an illness with no known scientifically proven cure. In my time serving at ELWA, as more health care workers became infected, fewer reported to duty, leading to a further shortage of personnel. I, too, experienced fear that I or my other colleagues would become infected as I watched several of my coworkers contract Ebola. In fact, I would sometimes have dreams at night that I had symptoms, prompting me to check my temperature in the middle of the night—sometimes several times. Each time I came into contact with bodily fluids of a patient with Ebola, I realized that I was putting myself at risk of contracting the disease. Despite this, I overcame my fears and continued to provide medical care to my patients, which included my own coworkers.

Fear of contracting Ebola was compounded by fear for personal safety; many times there were protests from local residents who were concerned about having a treatment center in their community. I was also cognizant that individuals who used to be child soldiers during Liberia's civil war earlier in the decade were now adults. I feared that the violence surrounding ELWA would soon escalate, making it impossible for patients to receive medical care. On my last day at ELWA, the treatment center was brimming with patients in every bed and there were a limited number of health care workers providing vital medical care while dozens of protesters stood outside chanting and shouting; the challenges in providing patient care were insurmountable.

Fear of contracting Ebola was compounded by fear for personal safety.

Fear of EVD is not anything new to me. As a child, I grew up in the Democratic Republic of the Congo and was there during the 1994 Ebola outbreak in Kikwit, 250 miles away from Kinshasa, where I lived. I recall clearly that the population in Kinshasa feared that the outbreak would spread; thus, Kikwit residents were quarantined. Little did I know, 20 years later, I would experience fear of Ebola in a completely different context.

But fear was not the only challenge in fighting the Ebola epidemic. Given the high case fatality rate, health care workers were repeatedly forced to face the death of those whose lives they had worked so hard to preserve. Every morning, I recall coming to the clinic and receiving the update that a patient my team had cared for had succumbed to EVD overnight. I remember vividly the mother of one of my patients looking into my eyes and pleading with me, “Please save my son. He is my only son. Don't let him die.” Ebola shows no such justice. Her son passed away two days later—his mother's worst fears were realized, and I was devastated.

As a physician, I have always aspired to provide compassionate care to each of my patients. Providing such compassionate care to patients with Ebola while wearing full personal protective equipment (PPE) proved to be extremely challenging. When health care providers are covered with PPE, it is difficult for patients to perceive their caregivers' emotions or to understand their body language. As there is no guarantee of recovery with Ebola, one of the few opportunities I had to demonstrate empathy to my patients was through my own verbal communication and nonverbal behavior, such as nodding or looking into the eyes of my patients with concern. Touching patients more than required for administering treatments, even while wearing full protective gear, was not advisable.

Providing compassionate care to patients with Ebola while wearing full personal protective equipment proved to be extremely challenging.

I often wondered if these communication barriers constructed by the PPE caused additional distress to patients. Already in physical isolation, afflicted with a deadly illness, and unable to witness the attitude and emotions of their health care provider, patients were mentally and emotionally isolated. To see the face of their health care provider obscured by a mask and hood only contributed to their trepidation. Furthermore, because of isolation requirements, many patients' sense of loneliness was magnified, as they died alone without family members nearby.

When my time at ELWA came to an end, I struggled to leave Monrovia knowing that the outbreak would not be contained any time soon. As a matter of fact, given the challenges of implementing infection control measures nationwide, I feared that the situation would soon escalate into a humanitarian emergency.

A myriad of factors have led to an inadequate response to this horrendous outbreak, from the lack of public health and health care delivery infrastructure to the persistence of unsafe burial practices and an environment of mistrust. Today, many Liberians believe either that Ebola does not exist or that patients with the disease are being experimented upon at clinics. In order to contain this outbreak, we must have a thorough understanding of cultural practices. And to overcome the mistrust, it is paramount that we use credible voices from Liberian communities to reinforce health promotion messages that are tailored to Liberian culture.

To overcome community mistrust, we must use credible community voices to reinforce health promotion messages that are tailored to the specific culture.

Despite these challenges, I have faith and am hopeful that the international community will come together and create a clear plan of action to prevent the loss of thousands of lives. I applaud the bravery of my Liberian colleagues, as well as those from Samaritan's Purse, Médecins Sans Frontières, and Serving in Mission, who have engaged Ebola from the beginning and continue to provide care despite many obstacles.

This is a unique opportunity for the international community to combine our technical expertise in public health, medicine, disaster management, and social science to better understand and address barriers that are preventing an effective response to the Ebola outbreak of 2014. It is also an opportunity to review how we can build the health infrastructure in low- and middle-income countries to prevent a disaster of this proportion from recurring.[Fig f01][Fig f02]

**Figure f01:**
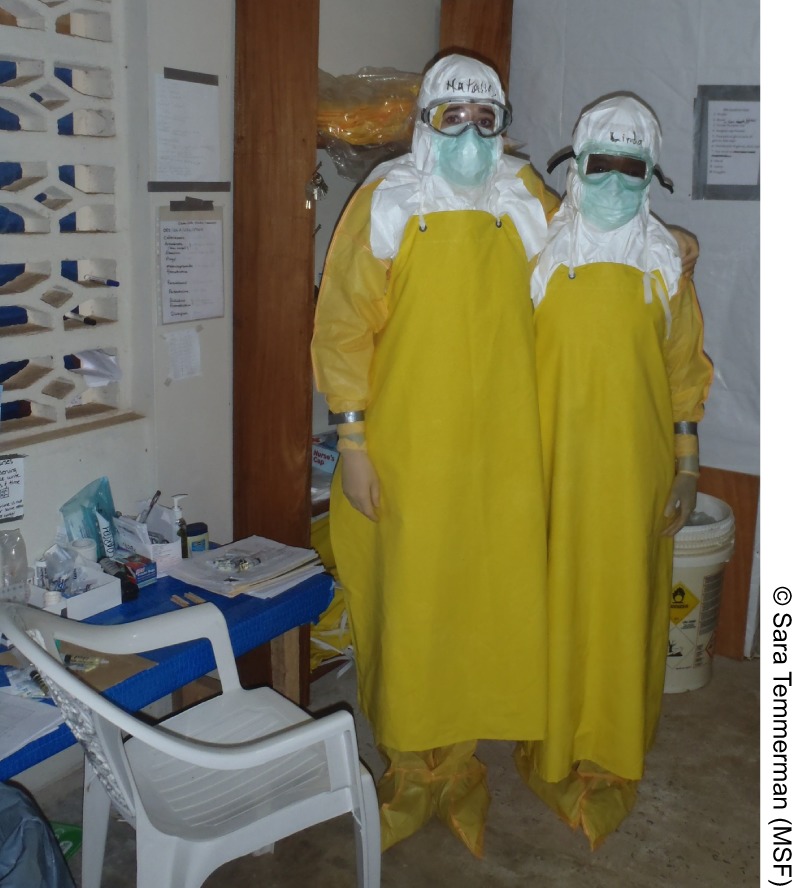
Dr. Linda Mobula and Dr. Nathalie McDermott wearing full personal protective equipment in the ELWA Ebola Case Management Center in Monrovia, Liberia.

**Figure f02:**
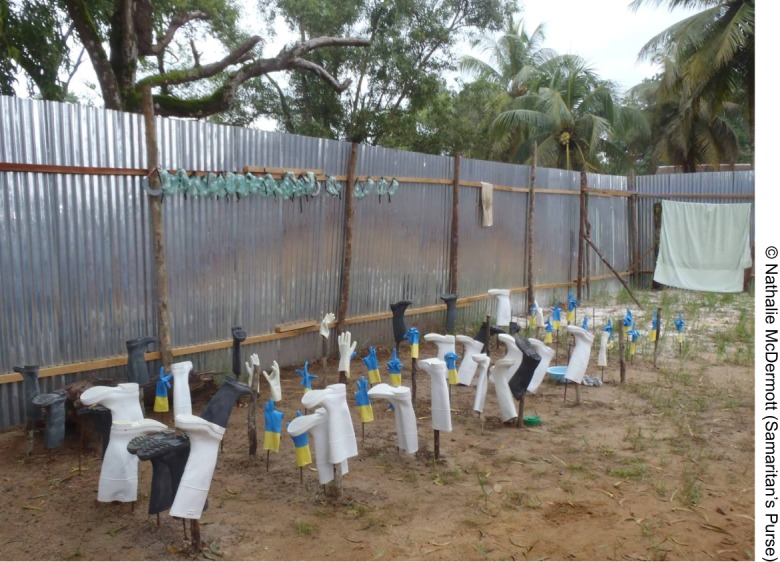
Gloves, boots, and goggles drying after decontamination with chlorine in the courtyard at the ELWA Ebola Case Management Center in Monrovia, Liberia.

## Notes

**First published online:** 2014 Oct 13
